# Age-related macular degeneration phenotypes are associated with increased tumor necrosis-alpha and subretinal immune cells in aged Cxcr5 knockout mice

**DOI:** 10.1371/journal.pone.0173716

**Published:** 2017-03-10

**Authors:** Hu Huang, Ying Liu, Lei Wang, Wen Li

**Affiliations:** 1 The Wilmer Eye Institute, Johns Hopkins University School of Medicine, Baltimore, MD, United States of America; 2 Aier Eye Hospital of Changsha, Hunan, China; 3 School of Ophthalmology, Central South University, Changsha, Hunan, China; Indiana University School of Medicine, UNITED STATES

## Abstract

The role of chemokine receptor in age-related macular degeneration (AMD) remains elusive. The objective of this study is to investigate the role of chemokine receptor Cxcr5 in the pathogenesis of AMD. Cxcr5 gene expression levels (mRNA and protein) are higher in the retina and retinal pigment epithelium (RPE) of aged C57BL/6 wild type mice than younger ones. Vascular and glial cells express Cxcr5 and its ligand Cxcl13 in mouse retina. Aged Cxcr5 knockout (^-/-^) mice develop both early and late AMD-like pathological features. White and yellow spots, which look like drusen in humans, were identified with fundscopic examination. Drusen-like sub-RPE deposits with dome-shaped morphology were characterized on the sections. RPE vacuolization, swelling, and sub-RPE basal deposits were illustrated with light and transmission electron microscope (TEM). TEM further illustrated degenerated and disorganized RPE basal infoldings, phagosomes and melanosomes inside RPE, as well as abnormal photoreceptor outer segments. Lipofuscin granules and lipid droplets in the subretinal space, RPE, and choroid were revealed with fluorescence microscope and oil-red-O staining. Increased IgG in RPE/choroid were determined with Western blots (WB). WB and immunofluorescence staining determined RPE zona occuldens (ZO)-1 protein reduction and abnormal subcellular localization. TUNEL staining, outer nuclear layer (ONL) measurement and electroretinogram (ERG) recording indicated that photoreceptors underwent apoptosis, degeneration, and functional impairment. Additionally, spontaneous neovascularization (NV)-like lesions develop in the subretinal space of aged Cxcr5^-/-^ mice. The underlying mechanisms are associated with increased subretinal F4/80^+^ immune cells, some of which contain RPE marker RPE65, and up-regulation of the multifunctional cytokine tumor necrosis factor-alpha (TNF-α) in RPE/choroid and retina. These findings suggest that Cxcr5 itself may be involved in the protection of RPE and retinal cells during aging and its loss may lead to AMD-like pathological changes in aged mice.

## Introduction

Age-related macular degeneration (AMD) is the most common cause of vision loss in the people aged 65 and older in the western world. Dry (atrophic) and wet (neovascular) AMD are the two major types of AMD. Dry AMD is characterized by retinal pigment epithelium (RPE) death; the hallmarks of wet AMD are choroidal neovascularization (CNV) and retinal angiomatous proliferation (RAP) [1]. While anti-VEGF is a treatment for wet AMD [2, 3], no treatment option is available for dry AMD. To discover new treatments for AMD, it is necessary to understand its etiology and pathophysiology. Drusen, subretinal or sub-RPE deposits are the early clinical hallmarks of AMD [4]. RPE death, photoreceptor degeneration, and CNV formation (in severe cases) occur in the later stages [5]. Both environmental triggers and genetic predisposition contribute to the disease development. The known environmental factors include cigarette smoking, light history, aging, diet, and race [6]. The identified susceptible genes include CFH [7–9], ABCA4 [10], ApoE [11], SOD1 [12] and Cx3cr1 [13] (see reviews for more details [14–17]). The interactions of environmental risk factors and susceptible gene variants can create state of oxidative stress, inflammation, and/or hypoxia [17–19]. Chronically, these pathological conditions cause damage to the photoreceptor/RPE/Bruch’s Membrane (BM)/choriocapillaris complex, resulting in the initiation and progression of AMD.

Chemokine receptors are hypothesized to play roles in the pathogenesis of AMD, because they are capable of regulating the migration of immune and inflammatory cells, which contributes to the initiation and development of AMD. Aged CC chemokine receptor (Ccr) 2^-/-^ and its ligand Ccl2^-/-^ mice developed AMD-like features [20]. Ccr3 was also a target in preventing CNV in wet AMD by pharmacological blockade and genetic deletion [21]. The T280M allele of Cx3cr1 gene was associated with increased incidence of AMD [13]. The homozygous mutation of Cx3cr1 gene led to the development of AMD-like features in senescent mice [22]. The Ccl2^-/-^/Cx3cr1^-/-^ double knockout (DKO) mouse line was an accelerated model of AMD [23]. However, recent studies raised some ambiguities about the role of these chemokine factors in the pathogenesis of AMD. Ccr3 did not mediate matrigel-induced CNV in rats or mice [24]. Deficiency of Ccl2 and Cx3cr1 did not appear sufficient to develop AMD phenotypes in aged mice [25, 26]. Additionally, AMD-like features in the Ccl2^-/-^/Cx3cr1^-/-^ DKO mice were caused probably by retinal degenerations as a result of rd8 mutations in the Crb1 genes (Crb1^rd8/rd8^ or rd8) [27–29]. Hence, the roles of chemokine receptors in the pathogenesis of AMD require further investigation.

Mammalian genome encodes about 20–30 chemokine receptors, which belong to the family of G-protein coupled receptor. These chemokine factors can be grouped into four classes based on their cognate ligands (C, Cc, Cxc, Cx3c). Cxcr5 is a receptor member of the Cxc sub-family [30]. It is expressed constitutively or inducibly by various cell types, such as inflammatory cells, RPE, and neuronal progenitors. The B-cells and T-cells that express Cxcr5 can be attracted to the inflammatory sites by Cxcl13 [31, 32]. In uveitis, Cxcr5^+^ dendritic cells (DC) were attracted to the retina due to retina-specific auto-antigens: inter-photoreceptor retinol-binding protein and s-antigen [33]. The cultured microglia expressed Cxcr5, which was up-regulated in the activated state by lipopolysaccharide [34, 35]. Monocyte-derived DC, other leukocytes in the eye [33], and skin-derived DC expressed Cxcr5 [36]. Cxcr5 in radial glial cells regulated the regenerative neurogenesis response in zebrafish brain [37]. RPE attracted B-lymphocytes to sub-RPE in the primary intraocular lymphoma via Cxcl13 expression [38]. In humans, neuronal progenitor cells expressing Cxcr5, when exposed to Cxcl13, migrated across the blood-brain barrier [39]. However, the role of Cxcl13-Cxcr5 signaling pathway in age-related changes, such as AMD, has not been explored.

In this study, we present evidence supporting that Cxcl13-Cxcr5 signaling pathway may play a protective role in the RPE and retinal cells of aged mice and loss of Cxcr5 may lead to the pathogenesis of AMD. Cxcr5 expression was up-regulated in the retina and RPE of the old WT mice compared with the younger ones. Vascular and glial cells expressed Cxcr5 and Cxcl13 in mouse retina. Aged Cxcr5^-/-^ mice developed both early and late AMD-like features, such as drusen-like sub-RPE deposits, RPE atrophy, photoreceptor apoptosis, lipid droplets, lipofuscin granules, and NV-like lesion in the subretinal space. The protein levels of tumor necrosis factor-alpha (TNF-α) were increased in the RPE/choroid and retina of aged Cxc5^-/-^ mice. Subretinal immune cells, some of which contained macrophage/microglia marker F4/80 and RPE marker RPE65, increased in aged Cxcr5^-/-^ mice.

## Materials and methods

### Animals

The Cxcr5^-/-^ (KO) mice [B6.129S2(Cg)-Cxcr5^tm1Lipp^/J] and C57BL/6 mice were bought from Jackson Laboratory. Aged C57BL/6 wild type (WT) control mice were obtained from National Institute of Aging (NIA, NIH). Both KO and WT mice were housed at the Wilmer Woods and Cancer Research Building Animal Facilities at Johns Hopkins Hospital, which are pathogen-free. The mice were fed with normal chow diets and provided with water ad libitum. The mice were anesthetized with ketamine hydrochloride (100mg/kg body weight) and xylazine (4mg/kg body weight). All the animal experiments in this study were specifically approved by the Institutional Animal Care and Use Committee (IACUC) of Johns Hopkins University School of Medicine and the guidelines of the Association for Research in Vision and Ophthalmology (ARVO) Statement for the use of animals in ophthalmic and vision research.

### PCR genotyping

The tail genomic DNA was prepared with the lysis reagent (DirectPCR; Wiagen Biotech, Los Angeles, CA). The PCR protocol for the Cxcr5 gene genotyping was based on the instructions provided by Jackson Laboratory. Briefly, PCR amplification was performed with three primers: CGG AGA TTC CCC TAC AGG AC (common), GAT CTT GTG CAG AGC GAT CA (WT reverse), and AAT TCG CCA ATG ACA AGA CG (mutant reverse).The PCR products were separated by gel electrophoresis on a 1.5% agarose gel. The mice with 241-bp PCR amplicon were the homozygous for the Cxcr5 mutation (Cxcr5^-/-^). The mice with 311-bp PCR amplicon were WT (Cxcr5^+/+^ or C57BL/6). The mice with both PCR amplicons were heterozygous (Cxcr5^+/-^). The Crb1 gene genotyping was performed as described previously [28, 40]. The mice with 244-bp PCR product were homozygous rd8 mutants (Crb1^rd8/rf8^, or rd8). The mice with 220-bp PCR product were Crb1 wild type (Crb1^wt/wt^). The mice with both PCR products were heterozygous (Crb1^rd8/+^).

### Fundus examination with the retinal-imaging microscope

Mice were anesthetized as described above. Pupils were dilated with 1% tropicamide. Cornea was protected with transparent gonioscopic gel. Fundus examination was performed with Micron III retinal-imaging microscope (Phoenix Research Labs, Inc., Pleasanton, CA).

### Immunofluorescence staining of sections and whole-mounts

Cryopreserved eye sections were air dried, fixed with 4% PFA, and then incubated with 10% goat serum (in PBS containing 0.25% Triton X-100) for 1 h. After washing with PBS, the specimens were incubated with the primary antibody (in PBS containing 0.05% Triton X-100 and 2% goat serum) at 4°C overnight. These primary antibodies were used: rabbit anti-Cxcr5/CD185 (1:200 dilution, Bioss, CA), rabbit anti-Cxcl13 (1:200, Thermo Fisher Scientific, Halethorpe, MD), rat anti-glutamine synthetase (GS, 1:200, BD Transduction Lab, Baltimore, MD), rabbit anti-active caspase 3 (1:200, cell signaling), mouse anti-CD11b (1:200, DSHB, Iowa), rat anti-GFAP (1:200, Thermo Fisher), rat anti-CD31 (1:200, BD Biosciences). After wash with PBS for 3 times (10min/time), specimens were incubated with the appropriate secondary antibodies conjugated with Alexa Fluor 488 or 594 (1:1000, Jackson Immuno Research Laboratories) at room temperature for 1 h. For staining with whole-mounts, the RPE/choroid tissues were incubated with 1% Triton-X 100 and dimethyl sulfoxide (DMSO; TD buffer in PBS) at 4°C overnight and then incubated with the primary antibody at 4°C overnight. The three primary antibodies (in TD buffer) were used: rat anti-ZO-1 (1:200 dilution, DSHB), rabbit anti-F4/80 (1:200, Sigma, St. Louis, MO), and mouse anti-RPE65 (1:200, Santa Cruz, Dallas, Texas). Following wash with PBS, the specimens were incubated with the appropriate secondary antibodies at 4°C overnight. DAPI acted as a counter stain. The stained specimens were observed and imaged with the Leica LSM510 scanning confocal microscopy system.

### Toluidine blue, Hematoxylin and Eosin (H&E) staining

One-micron semithin sections were cut with a Reichert Ultracut S microtome (Leica Microsystems Inc, IL, USA), stained with 1% toluidine blue, and examined with a light microscope. For H&E staining, the sections were incubated for 1 min with Harris Hematoxylin (Sigma) and washed for 5 min with running tap water. After staining with 0.5% Eosin for 20 s and washing with distilled water, the specimens were dehydrated with a series of gradient ethanol and then mounted with xylene-based mounting media for microscope examination.

### Oil Red O staining

Cryopreserved sections were incubated with 0.5% Oil Red O (dissolved in 1,2-iso-propanol) for 15 min at 60°C, differentiated in 85% 1,2-iso-propanol for 5 min, and rinsed in distilled water twice (5 min/time). The sections were then mounted in aqueous mounting media for photodocumentation.

### Transferase-mediated dUTP nick-end labeling (TUNEL) assay

Apoptotic cells in retinal tissue were detected by TUNEL assay using ApopTag Red In Situ Apoptosis Detection Kits (Millipore, Billerica, MA) according to the manufacturer’s protocols. Briefly, cryopreserved eye sections were collected and fixed as described above. Tissue sections were post-fixed with the mixture of ethanol: acetic acid (2:1) at -20°C for 5 min, washed three times with PBS and then immerged in equilibration buffer for 1min. After removal of the equilibration buffer, the sections were incubated with TUNEL working strength TdT enzyme reaction mixture at 37°C for 1 h, followed by incubation with anti-digoxigenin (conjugated with rhodamine) at room temperature for 30 min. DAPI was used to counterstain. TUNEL-positive nuclei were visualized and imaged with the Leica LSM510 scanning confocal microscopy system.

### Transmission electronic microscopy (TEM)

Eyes were enucleated and fixed with 2.5% glutaraldehyde and 2.5% paraformaldehyde (in 0.1M cacodylate buffer, pH 7.4) for TEM. The anterior segment and vitreous humor were removed. The eyecups with the retina, RPE and choroid were fixed with 1% osmium tetroxide in 0.1M cacodylate buffer (pH7.4). The fixed eyecups were dehydrated with gradient alcohols and embedded in Poly/Bed 812 resin. 70-nanometer ultrathin sections were cut with a Leica EM UC 7 microtome and stained with uranyl acetate and lead citrate. The stained specimens were analyzed with a Hitachi H-7600 TEM instrument (Hitachi Co. Ltd., Tokyo, Japan).

### Real-time quantitative polymerase chain reaction (QPCR)

Total RNA from mouse tissues (retina or RPE/choroid) was isolated using an RNeasy Mini Kit (Qiagen, Valencia, CA), with on-column DNA digestion by RNase-free DNase (Qiagen). cDNA was synthesized using a High Capacity cDNA Reverse Transcription kit (Applied Biosystems Inc., Foster City, CA). Each real-time PCR reaction consisted of 9 μl of cDNA template, 10 μl of Taqman Fast Universal PCR Master Mix (ABI), and 1 μl of ABI's pre-mixed primers and Taqman MGB (minor groove binder) probe set on a StepOne Plus Thermocycler (ABI) for 40 cycles (94°C for 15 s, 60°C for 20 s) after an initial 20 s of incubation at 94°C. The percentage change in expression of each gene was calculated using the comparative Ct method, with cyclophilin A (PPIA) as internal control. Primer sequences for Cxcr5 were catgggctccatcacataca (forward) and gtgcctctccaggattacca (reverse).

### Western blots (WB) analysis

WB was performed as previously described with some modifications [41]. Dissected mouse retina or RPE/choroid was sonicated in cold RIPA buffer containing FAST Protease Inhibitor (Sigma). Protein content from the retina or RPE/choroid was quantified using the Bio-Rad DC Protein Assay kit (Hercules, CA). 5–20 μg protein per lane was separated by 4–12% Bis-Tris SDS-PAGE (Life Technologies) and transferred to 0.2 μm pore size nitrocellulose membranes. Membranes were blocked with 5% non-fat milk at room temperature for 1 h and then incubated overnight at 4°C with the following primary antibodies: anti-Cxcr5 (1:500, Bioss), anti-ZO-1 (1:500, DSHB), anti-TNF-α (1:500, Janssen, PA), anti-GAPDH (1:2500, Abcam), and anti-β-actin (1:2500, Cell Signaling) followed by incubation with horseradish-peroxidase (HRP)-conjugated secondary antibody (1:4000; Cell Signaling) for 1 h at room temperature. Signal was detected by enhanced chemiluminescence (ECL) using SuperSignal West Pico or Femto kit (Thermo Scientific) and GE Healthcare's ImageQuant LAS 4010 Digital Imaging System (Pittsburgh, PA). Densitometry was performed using Image J (NIH, Bethesda, MD).

### Electroretinography (ERG)

ERG recording was performed with a UTAS system (LKC Technologies, Gaithersburg, MD) on 17-month-old WT and KO mice. The ERG procedures were in accordance with the standard protocols and the manufacturer’s manual. Briefly, mice were dark-adapted overnight prior to scotopic ERG. After the pupils were dilated with a 0.1% Tropicamide, the mice were anesthetized and placed on a platform. A gold loop filament was contacted with the cornea and acted as the positive electrode. One needle was subcutaneously inserted into the anterior scalp between the ears and served as the reference electrode. Another needle was inserted underneath the skin near the tail and served as the ground electrode. A LKC ganzfeld illuminator stimulated the eyes for electrical impedance. Ten scotopic ERGs were recorded for each of the six intensity levels of flashlight ranging from −30 to 10dB with an 8dB interval. The 10 values were averaged using the UTAS signal averaging system. The amplitude of a-wave from scotopic ERG was measured from the baseline to the negative peak, which demonstrated the functions of rod photoreceptors. The amplitude of b-wave was measured from the trough of a-wave to the peak of b-wave, indicating the functions of inner retina.

### Statistical analysis

Statistical comparisons were made using analysis of variance (ANOVA) or a linear mixed model (for ERG data) [42]. The non-parametric Mann-Whitney U-test was performed to determine the significance level between two groups (for quantification data with Real-time PCR and WB). P<0.05 was designated as being statistically significant.

## Results

### Increased Cxcr5 gene expression in the retina and RPE of aged C57BL/6 WT mice

**Fig 1:**
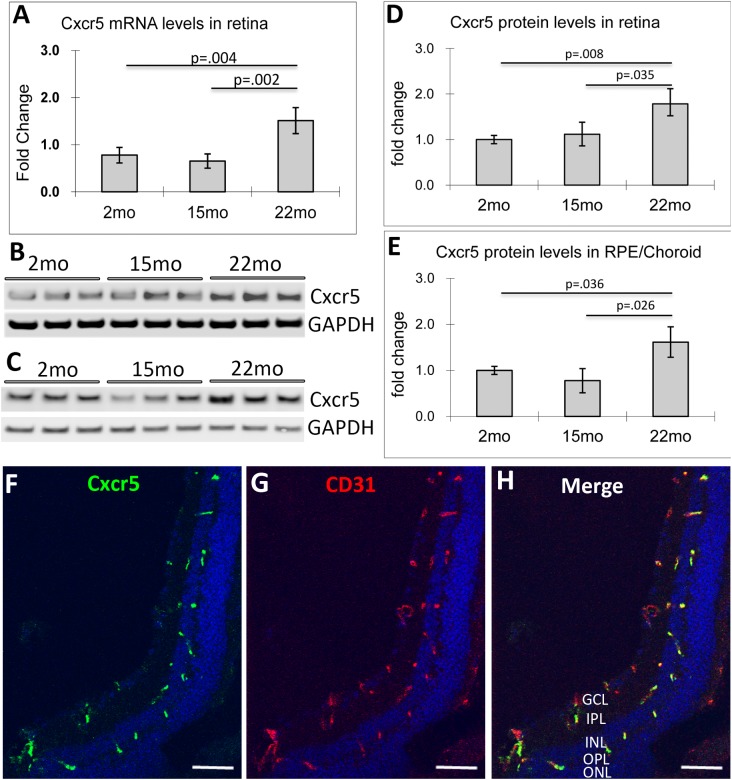
The increased Cxcr5 gene expressions (mRNA and protein) in the retina and RPE of aged mice. **(A)** Real-time PCR for Cxcr5 mRNA levels in retina. The normalized value at the age of 2 months acted as the baseline, with which the other two points (15 and 22 months) compared. The results were expressed as the mean fold change against the baseline value (± SD, n = 4). **(B and C)** Western blots (WB) for Cxcr5 protein levels in retina (**B**) and RPE/choroid (**C**). (**D & E**) WB Quantification of retina (**D**) and RPE/choroid (**E**). The normalized optical density ratio of Cxcr5 and GAPDH at the age of 2 months acted as a baseline value, with which the other two pointes (15 and 22 months) compared. The results were expressed as the mean fold change against the baseline value (± SD, n = 3). (**F and G**) Double Immunofluorescence staining of Cxcr5 (**F**) and CD31 (**G**) in mouse retina. The merged image (**H**) demonstrated the co-localization of Cxcr5 and CD31. The sections were prepared from adult (2 months old) C57BL/6 wild type mice. GCL: ganglion cell layer; IPL: Inner plexiform layer; INL: inner nuclear layer; OPL: outer plexiform layer; ONL: outer nuclear layer. Scale bar: 50 μm.

In one previous report, we compared the cytokine profile between the CNV, which was induced by laser injury in adult C57BL/6 mice, and periphery tissue (without or little NV) using the method of laser capture microdissection (LCM) [43]. We found that Cxcr5 gene expression level was higher in the central CNV than the periphery, suggesting the co-relation of Cxcr5 with NV in the laser-induced CNV model [44]. In the present study, we further examined Cxcr5 gene expression in the retina and RPE/choroid of the old and young C57BL/6 WT mice. QPCR indicated that Cxcr5 mRNA levels were increased 1.9 fold in retina of the 22-month-old mice as compared to 2-month-old mice (p = 0.004, n = 4), and 2.9 fold to the 15-month-old mice (p = 0.002, n = 4) (Fig 1A). Using an anti-Cxcr5 antibody (see S1 Fig for its specificity), we performed WB and densitometry analysis. The results revealed that Cxcr5 protein levels were increased i) 1.8 fold in the retina of 22-month-old mice as compared to 2-month-old mice (n = 3, p = 0.008) and 1.4 fold to the15-month-old mice (p = 0.035, n = 3) (Fig 1B & 1D), and ii) 1.6 fold in RPE/choroid of the 22-month-old mice as compared to 2-month-old mice (p = 0.036, n = 3), and 2.1 fold to 15-month-old mice (p = 0.026, n = 3) (Fig 1C & 1E). The Cxcl13 mRNA level, however, was not significantly increased in retina along aging (data not shown).

Immunofluorescence staining was performed to examine retinal cells that express Cxcr5 and Cxcl13. Immunoreactivity for Cxcr5 localized in outer plexiform layer (OPL), inner nuclear layer (INL), inner plexiform layer (IPL), and ganglion cell layer/nerve fiber layer (GCL/NFL) in mouse retina (Fig 1F). Double staining with anti-Cxcr5 and anti-CD31 confirmed Cxcr5 expression in vascular cells in the adult (2 months) mouse retina. In aged mouse retina (e.g., 22 months), glial cells predominantly expressed Cxcr5, which were indicated by co-localization of Cxcr5 and lectin, CD11b, or GFAP (S2 Fig). Immunoreactivity for Cxcl13 localized in OPL, IPL, and GCL/NFL in adult mouse retina. The cellular distribution of Cxcl13 looked like vasculatures in OPL and Mϋller glial cells (MGC) in IPL and GCL/NFL (S3A Fig). Cxcl13 expression in vascular cells was reported previously [45]. MGC identity was identified with the marker GS, which co-localized with Cxcl13 in the inner retina (S3C–S3E Fig). In aged wild type mouse retina (15 months), Cxcr13 was also expressed by some other cell types, especially in the inner part of inner nuclear layer (INL), where amacrine cells reside (S3C Fig). In aged Cxcr5^-/-^ mouse retina (15 months), Cxcl13 had more profound expression in the process of MGC (S3B Fig).

### Fundus abnormalities in aged Cxcr5^-/-^ mice

**Fig 2:**
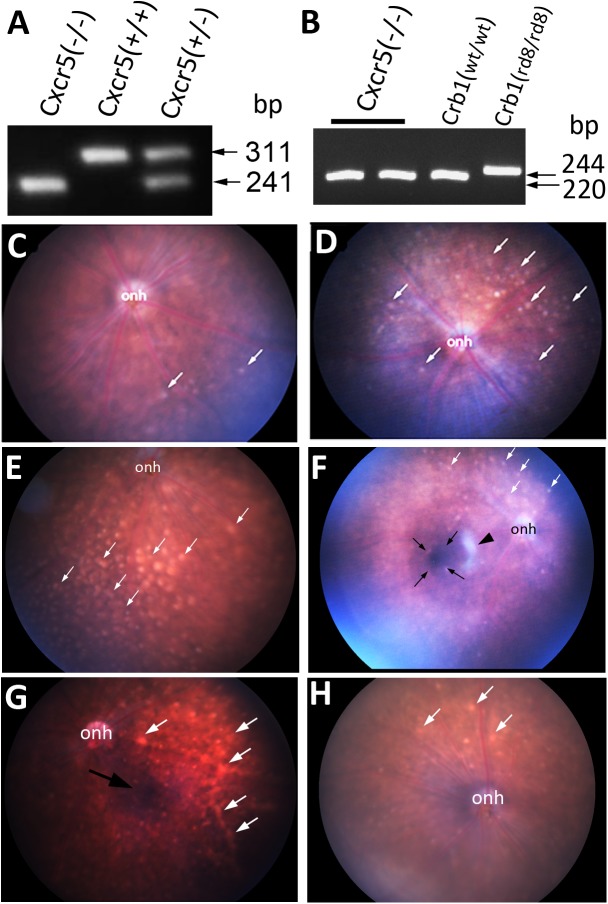
Fundus abnormalties in aged Cxcr5^-/-^ mice. (**A**) PCR genotyping results for Cxcr5 gene. (**B**) PCR genotyping results for Crb1 gene.(**C-H**) Representative fundus images of Cxcr5^-/-^ mice at the ages of 5 months old (**C**), 9 months old (**D**), 12 months old (**E**), 17 months old (**F**), 22 months old (**G**), and the 22-month-old C57BL/6 wild type (WT) control mice (**H**). White arrows (**C-H**) denoted drusen-like spots. Black arrows (**F & H**) denoted the possible hyperpigmentation. Black arrowhead (**F**) denoted the possible hypopimgmentation. onh: opitcal nerve head. Four to eight mice from each group were used for retinal fundus imaging analysis.

Increased Cxcr5 gene expression along aging and its retinal cellular distribution suggest that Cxcr5 itself may play a role in the homeostasis of aged eye and its loss may be implicated in age-related pathologies, such as AMD. To explore this possibility, Cxcr5^-/-^ mice were obtained from the Jackson Lab. The genotypes of Cxcr5 heterogeneous and homogenous mutations were confirmed by PCR (Fig 2A). As rd8 mutation is known to contaminate the founder mouse lines from some vendors, leading to eye phenotypes [28, 40], Crb1 gene genotyping analysis was performed to determine if the Jackson Cxcr5^-/-^ mice had the rd8 mutation. The results corroborated that this mouse line did not have the rd8 gene mutation (Fig 2B).

Fundscopic examination with Micron III imaging system was performed to see whether there are fundus abnormities in aged Cxcr5^-/-^ mice. Numerous white spots, which looked like drusen in human AMD, were present in fundus of aged (9 monthsold) Cxcr5^-/-^ mice, but only a few white spots were observed in adult (5 months old Cxcr5^-/-^ mice, (Fig 2C & 2D). These apparent deposits became worse as the mice got older (Fig 2E–2G). For example, the spots enlarged; some of them fused together. Aged Cxcr5^-/-^ mice appeared to develop RPE abnormities, as indicated by the hyper-/hypo-pigmented RPE areas. The aged WT control mice, however, developed no or very few spots (Fig 2H).

### Histopathological and ultra-structural changes in the RPE and BM of aged Cxcr5^-/-^ mice

**Fig 3:**
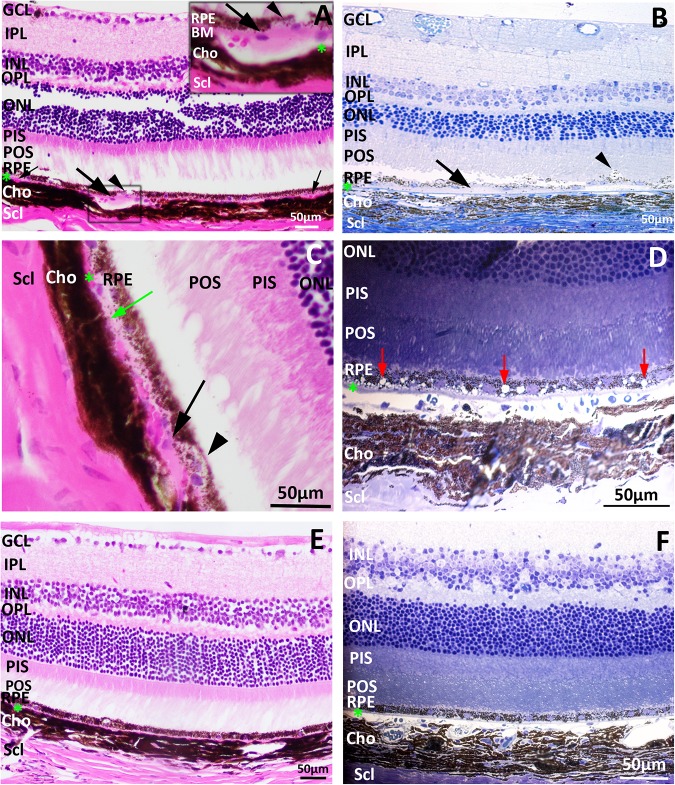
Drusen-like, sub-RPE deposition, and RPE vacuolization in aged Cxcr5^-/-^ mice. Retinal cross sections were prepared from 17-month-old Cxcr5^-/-^ mice (**A**-**D**) and C57BL/6 WT control mice (**E** and **F**). Cryo-preserved sections were stained with H & E (**A**, **C**, and **E**). Plastic sections were stained with toluidine blue (**B**, **D**, and **F**). (**A**) Drusen-like deposits. Large arrows indicated the dome-shaped drusen-like deposits between the RPE and Bruch’s membrane (BM, green asterisks). Small arrows indicated the two small drusen-like deposits. Inset showed the drusen-like deposits in the box. Arrowheads indicated the degenerated RPE. (**B**) Sub-RPE deposits (arrows) and RPE distention into the subretinal space (arrowhead). (**C**) BM degeneration (green arrow), BM thickening (black arrow), and RPE atrophy (arrowhead). (**D**) Numerous vacuoles in RPE (red arrows). (**E and F**) H & E-/ toluidine blue-stained sections from the WT control mice. Three to five mice from each group were used. GCL: ganglion cell layer; IPL: Inner plexiform layer; INL: inner nuclear layer; OPL: outer plexiform layer; ONL: outer nuclear layer; PIS: photoreceptor inner segment; POS: photoreceptor outer segment; RPE: retinal pigment epithelium; Cho: choroid; Scl: sclera. Green asterisk: Bruch’s membrane (BM).

Histopathological changes in the RPE and BM of aged Cxcr5^-/-^ mice was examined with the sections stained with toluidine blue or H & E. As shown in Fig 3A, the drusen-like deposits were present in the sub-RPE between RPE and BM. The dome-shaped appearance, focal deposition, and sub-RPE location suggested these deposits in aged Cxcr5^-/-^ mice were similar to the drusen seen in human AMD. In addition to drusen-like deposits, abnormal BM and sub-RPE basal depositions were also observed in aged Cxcr5^-/-^ mice: Fig 3B showed extended sub-RPE basal deposits; Fig 3C showed abnormal BM in aged Cxcr5^-/-^ mice (degeneration and thickening). Drusen-like deposits, abnormal BM, and sub-RPE basal depositions were associated with RPE degeneration (Fig 3A and 3C) or distension (Fig 3B). Additionally, many RPE cells were vacuolated in aged Cxcr5^-/-^ mice (Fig 3D). However, it is important to note that RPE vacuolization in human AMD is actually quite rare and not considered a phenotypic feature of the disease [46], though it has been reported in mouse models of AMD. No apparent abnormalities were observed in the RPE and BM of aged WT control mice and adult Cxcr5^-/-^ mice (Fig 3E and 3F).

**Fig 4:**
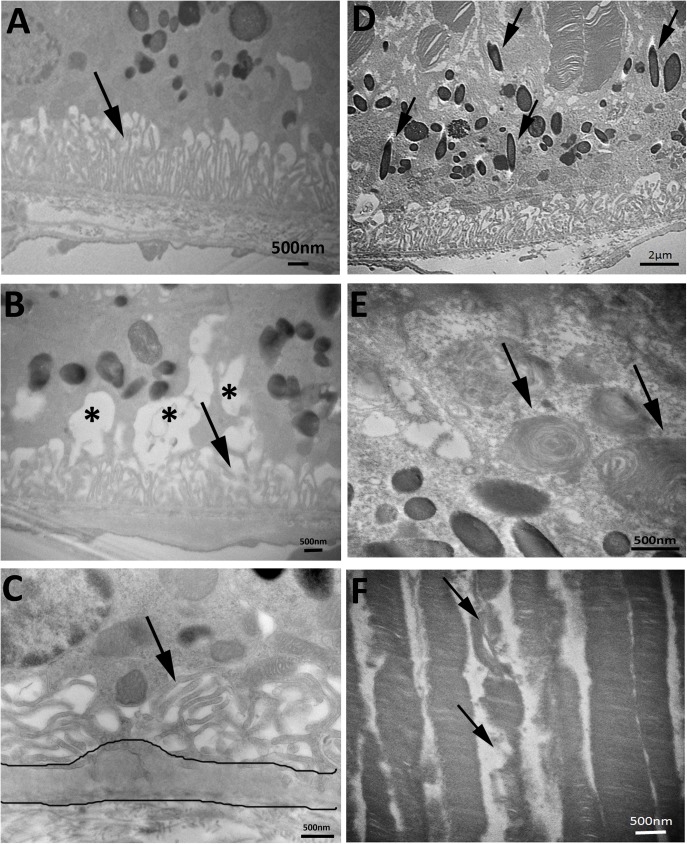
RPE ultra-structural changes in aged Cxcr5^-/-^ mice. **(A)** RPE of the adult (5 months old) Cxcr5^-/-^ mice. Arrows indicated normal RPE basal infolding. **(B)** RPE vacuoles with membranous debris inside (asterisks) and reduced basal infolding (arrow)**. (C)** Disorganized basal infolding (arrows) and sub-RPE basal deposits (between the two lines). **(D)** Melanosomes in basal cytoplasm of RPE (arrows). **(E)** Phagosomes with photoreceptor outer segments (arrows) in RPE. **(F)** Abnormal photoreceptor outer segments (arrows). Representative images from four mice were used for the demonstration of each abnormality.

TEM analysis was applied to reveal ultra-structural changes of the RPE abnormalities observed with light microscope. RPE of adult (5 months old) Cxcr5 KO mice appeared normal: well-organized basal infolding, no vacuoles, nor sub-RPE deposits (Fig 4A). Aged KO mice, however, developed various abnormities. 1) Copious vacuoles with membranous debris were present in RPE (Fig 4B). 2) Electron dense material was deposited on the basal cytoplasm of RPE and associated with disorganized and reduced basal infolding (Fig 4C). 3) Melanosomes were frequently present in the basal cytoplasm of KO mice (Fig 4D), but were apically localized in the WT control mice of same age. 4) Phagosomes with photoreceptor segments were present inside RPE (Fig 4E). 5) Abnormal photoreceptor segments have also been identified (Fig 4F).

### Increased lipid droplets, lipofuscin granules, and IgG in aged Cxcr5^-/-^ mice

**Fig 5:**
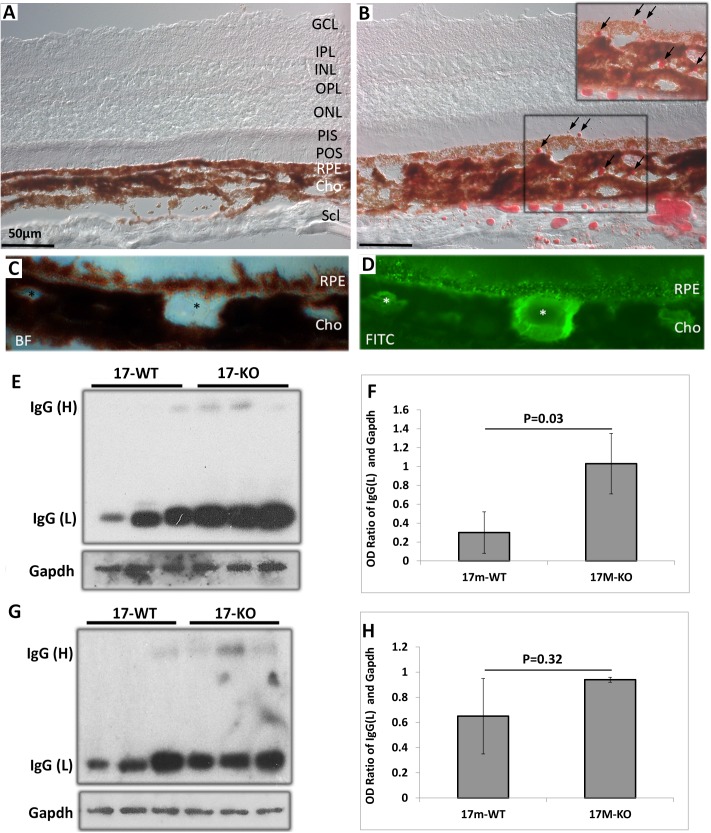
lipid droplets, lipofuscin granules, and increased IgG in aged Cxcr5^-/-^ mice. 17-month-old C57BL/6 WT mice and Cxcr5 KO mice were used. (**A & B**) Oil Red O staining of the WT mice (**A**) and the KO mice (**B**). Arrrows (**B**) indicated lipid droplets in sub-retinal space, RPE, and choroid. (**C and D**) Bright-field micrograph and lipofuscin granules in RPE and choroiod. BF: bright field channel. FITC: fluorescin channel. (**E and G**) WB results of RPE/choroid (**E**) and retina (**G**). Anti-mouse IgG secondary antibody was incubated with protein blots, which were not incubated with any primary antibody, to examine the endogenous IgG. Gapdh acted as protein loading controls. The upper bands were about 55KD IgG heavy chain or IgG (H). The lower bands were about 25KD IgG light chain or IgG (L). (**F and H**) IgG (L) WB quantificationfor RPE/choroid (**F**) and retina (**H**). GCL: ganglion cell layer; IPL: Inner plexiform layer; INL: inner nuclear layer; OPL: outer plexiform layer; ONL: outer nuclear layer; IS: inner segment; OS: outer egment; RPE: retinal pigment epithelium; Cho: choroid; Scl: sclera.

17-month-old WT and KO mice were examined for lipofuscin deposits and lipid droplets. Oil-red-O (ORO) staining revealed that lipid droplets were present in the subretinal space, RPE, and choroid of the KO mice, but not the WT mice (compare Fig 5A and 5B). It is worth noting that the ORO staining pattern in aged KO mice looks different from that observed in aged human BM, which is more diffuse than focal [47]. Lipofuscin granules were deposited in RPE and choroid, particularly in the cavities of choriocapillaris (Fig 5C & 5D). Furthermore, WB and densitometry analyses revealed that IgG was significantly increased in RPE/choroid (but not retina) of the KO mice, compared with the age-matched WT control mice (Fig 5E–5H).

### RPE atrophy in aged Cxcr5^-/-^ mice

**Fig 6:**
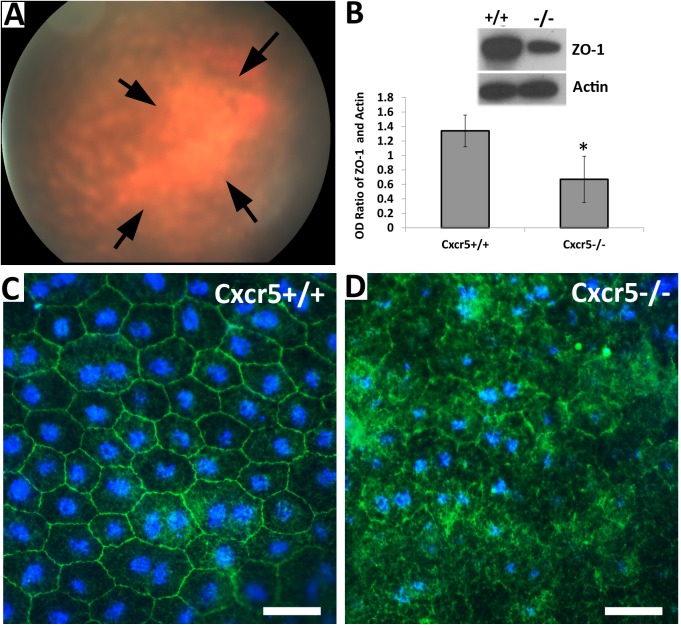
RPE atrophy in aged Cxcr5^-/-^ mice. **(A)** Retinal fundus image displayed the changes suggestive of geography atrophy in the 17-month-old Cxcr5^-/-^ mice. Arrows indicated the large demarcated atrophic RPE area. (**B**) Western blots (top) showed that ZO-1 protein was reduced in aged Cxcr5^-/-^ mice, compared with the age-matched WT mice. Densitometry analysis (bottom) indicated ZO-1 protein was significantly reduced in the KO as compared to the WT. The values were the mean optical density (OD) ratio of ZO-1 and Actin (± SD; n = 3). * denoted p<0.05. (**C and D**) Immunofluorescence (IF) staining results revealed the ZO-1 (+) hexagonal grid was well organized in the RPE/choroid whole-mounts of the WT mice (**C**), but disorganized in the Cxcr5^-/-^ mice (**D**). Three 17-month-old C57BL/6 WT mice and Cxcr5^-/-^ mice were used for IF analysis.

17-month-old Cxcr5^-/-^ mice were further analyzed for RPE atrophy. Funduscopic examination found the large sharply demarcated atrophic RPE area or geographic atrophy (GA, Fig 6A). The tight junction ZO-1 protein in RPE was significantly reduced in aged Cxcr5^-/-^ mice, compared with the age-matched WT mice (Fig 6B). Further immuofluorescence staining showed that ZO-1 was localized along with the plasma membrane of RPE and the hexagonal grid was well organized on RPE surface of the WT mice (Fig 6C). However, ZO-1 was aberrantly localized in RPE of the aged Cxcr5^-/-^ mice (Fig 6D).

### Photoreceptor degeneration in aged Cxcr5^-/-^ mice

**Fig 7:**
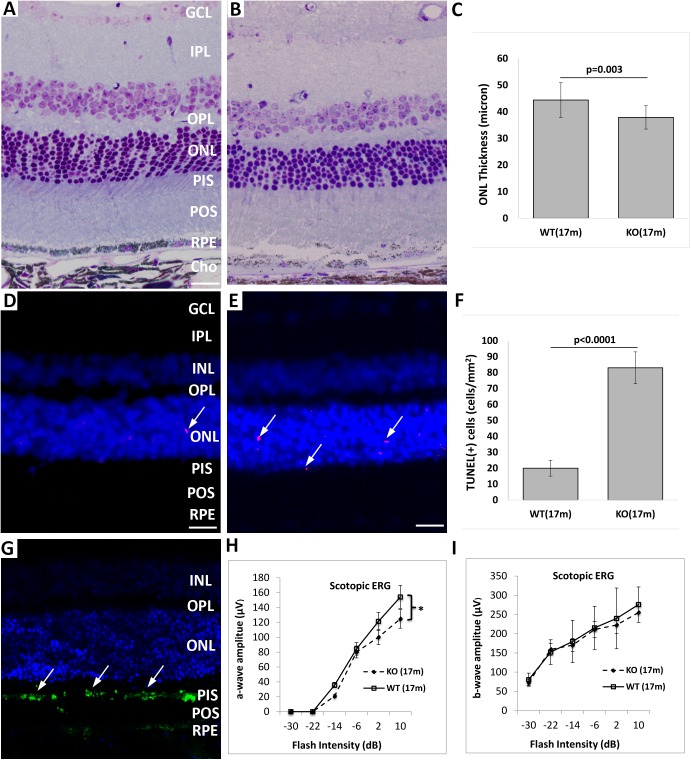
Photoreceptor degeneration in aged Cxcr5^-/-^ mice. 17-month-old C57BL/6 WT mice and Cxcr5 KO mice were used. (**A and B**) Representative toludin blue-stained images of the WT mice (**A**) and the KO mice (**B**). (**C**) Quantification of ONL thickness. The superior retinal areas that were near optical nerve head approximately 100 micron were used for ONL thickness measurement. The values were the mean (± SD; n = 10). (**D and E**) Representative TUNEL staining images of the WT mice (**C**) and the KO mice (**D**). Arrows indicated the TUNEL (+) cells in ONL layer. (**F**) Quantification of TUNEL (+) cells (mean ± SD; n = 6). (**G**) Representative active Caspase-3 staining images of the KO mice. The merged picture with DAPI staining showed the localization of the active caspase-3 in photoreceptor inner segments. Arrows indicated active Caspase-3 staining signals. (**H and I**) A-wave (H) and b-wave (I) amplitudes of scotopic ERG. The values were the mean (± SD; n = 10). * denoted p <0.05. GCL: ganglion cell layer; IPL: Inner plexiform layer; INL: inner nuclear layer; OPL: outer plexiform layer; ONL: outer nuclear layer; PIS: photoreceptor inner segment; POS: photoreceptor outer segment; RPE: retinal pigment epithelium.

There was about 20% reduction in the ONL thickness of 17-month-old Cxcr5 KO mice, compared with WT control mice: 44.4 ± 6.5 micron for the WT and 37.9 ± 4.4 micron for the KO (p = 0.003, n = 5) (Fig 7A, 7B and 7G). Photoreceptor nuclear layers were also reduced: 10–12 layers for the WT mice and 7–9 layers for the KOs (p = 0.02, n = 5). TUNEL stain indicated that apoptotic cells were significantly higher in the KO mice than the WT controls: 20 ± 5 TUNEL (+) cells/mm^2^ for the WT mice and 83 ± 10 TUNEL (+) cells/mm^2^ for the KOs (Fig 7C, 7D and 7F). Active Caspase-3 stain further confirmed that photoreceptors underwent apoptotic cell death in aged Cxcr5^-/-^ mice (Fig 7E). A-wave measurement of Scotopic ERG recording indicated that rod photoreceptor function was impaired in response to high flash intensity (10 dB) in aged Cxcr5^-/-^ mice, compared with the age-matched WT mice(Fig 7H). There were not significantly differences in b-wave amplitude between the aged WT and KO mice (Fig 7I).

### Increased immune cells in the subretinal space of aged Cxcr5^-/-^ mice

**Fig 8:**
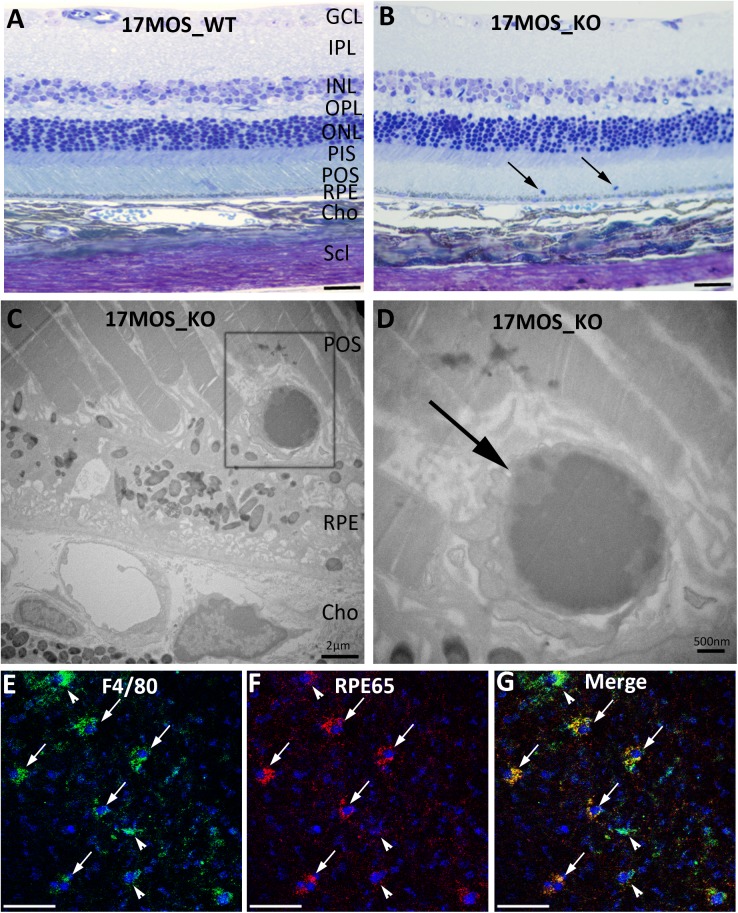
Increased subretinal immune cells in aged Cxcr5^-/-^ mice. (**A & B**) Representative toluidine blue-stained sections of 17-month-old C57BL/6 WT mice (**A**) and Cxcr5 KO mice (**B**). Arrows indicated the subretinal immune cells in the KO mice. (**C & D**) Transmission electron microscope (TEM) image of the photoreceptor outer segment/RPE/choroid interface (**C**) and higher-resolution TEM image of the subretinal immune cells in the box (**C**). Arrow (**D**) indicated the subretinal immune cells. (**E-G**) Dual labeling of macrophage/microglia marker F4/80 and RPE marker RPE65 with the RPE/choroid whole-mounts from the KO mice: F4/80 (**E**), RPE65 (**F**), and the merged (**G**). Arrows indicated the cells that were immune positive for both markers. Arrowheads indicated the cells that were immune positive for F4/80, but negative or had less staining intensity for RPE65. Scale bar: 50μm. GCL: ganglion cell layer; IPL: Inner plexiform layer; INL: inner nuclear layer; OPL: outer plexiform layer; ONL: outer nuclear layer; PIS: photoreceptor inner segment; POS: photoreceptor outer segment; RPE: retinal pigment epithelium; Cho: choroid; Scl: sclera.

Cxcr5 can regulate the trafficking of immune cells in lymphatic system [31] and neuronal progenitors in central nervous system [39]. We hypothesize that Cxcr5 regulates the migration and infiltration of immune and/or inflammatory cells in the eye. Light microscopic examination found that the increased cell accumulations in the subretinal space of aged Cxcr5^-/-^ mice. This was, however, rarely observed in the WT mice of same age (Fig 8A & 8B). Higher-resolution TEM images showed that the infiltrated cells had the appearance of immune and inflammatory cells (Fig 8C & 8D). Immunofluorescence staining with the RPE/choroid whole-mounts revealed that the cells were immunopositive for F4/80, a maker for microglia/macrophage. Further double staining revealed that a portion of these immune and inflammatory cells contained RPE cell marker RPE65 (Fig 7E–7G). Why these sub-retinal cells contain both macrophage/microglia marker and RPE marker needs to be further investigated.

### Increased TNF-α protein in the RPE/choroid and retina of aged Cxcr5^-/-^ mice

**Fig 9:**
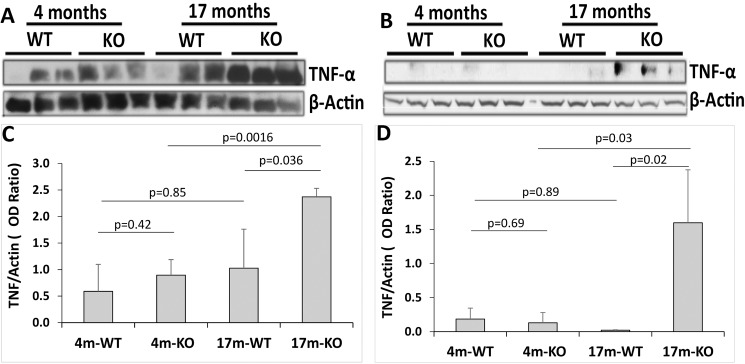
Increased protein levels of TNF-α in the RPE/choroid and retina of aged Cxcr5^-/-^ mice. (**A and B**) Western blots (WB) of TNF-α and β-actin with the RPE/choroid (**A**) and retina (**B**). The RPE/Choroid and retinal protein samples from three individual mice were used for each group. Protein blot was first probed by anti-TNF-α antibody. After stripping and washing, the same blot was re-probed by anti-β-actin antibody. (**C and D**) WB quantification of RPE/choroid (**C**) and retina (**D**). The results were the mean optical density (OD) ratio of TNF-α and β-actin (± SD; n = 3). 4/17m-WT = 4/17-month-old C57BL/6 wild type mice. 4/17m-KO = 4-month-old Cxcr5 knockout mice.

Finally, we examined the protein levels of TNF-α, which is a multifunctional pro-inflammatory cytokine and plays a critical role in apoptosis and necroptosis [48, 49]. The total proteins were prepared from the RPE/choroid and retina proteins of 4-month-old WT, 4-month-old KO, 17-month-old WT, and 17-month-old KO and then were utilized for WB analyses. As shown in Fig 9A, TNF-α protein levels in RPE/choroid varied from undetectable to moderate among three individual mice of the first three groups, but they were more consistent and robust in mice of the17-month-old KO group than the other three. Densitometry analysis (Fig 9C) indicated that the protein levels of TNF-α were not significantly different between the 4-month-old WT and the 4-month-old KO (the mean OD ratio of TNF-α and β-actin: 0.59 ± 0.51 for the WT; 0.89 ± 0.29 for the KO; p = 0.42; n = 3) as well as between the 4-month-old WT and the 17-month-old WT (the mean OD ratio: 1.03 ± 0.73 for the WT; p = 0.85; n = 3). The difference was significant between the 4-month-old KO and the 17-month-old KO (the mean OD ratio: 2.37 ± 0.16 for the 17-month-old KO; p = 0.036; n = 3) as well as between the 17-month-old WT and the 17-month-old KO (p = 0.0016; n = 3). Total protein abundance was evidently lower in retina than RPE/choroid: WB did not or barely detected TNF-α in the young WT and young KO groups as well as the old WT group, but TNF-α protein was increased in the old KO group (Fig 9B). Further densitometry analysis confirmed the significant difference between the old KO and the other three animal groups: the mean OD ratio of TNF-α and β-actin was 0.19 ± 0.16 for young WT, 0.13 ± 0.15 for old WT, 0.02 ± 0.008 for young KO, and 1.60±0.78 for young KO (p = 0.002 vs. young KO; p = 0.03 vs. old WT),.

## Discussion

The chemokine receptor Cxcr5 has been received little attention in the eye. Our study provides insights into the role of Cxcr5 in the RPE and retinal cells of aged eye and the relation of its gene deletion to the pathogenesis of AMD. Cxcr5 gene expressions (mRNA and protein) were increased in the retina and RPE/choroid of old WT mice as compared to the younger ones. Vascular and glial cells expressed Cxcr5 and its ligand Cxcl13 in mouse retina. Drusen-like sub-RPE deposits were present in aged Cxcr5^-/-^ mice. Lipofuscin granules and lipid droplets were deposited in the subretinal space, RPE and choroid of aged Cxcr5^-/-^ mice. Subretinal immune and inflammatory cells were increased in aged Cxcr5^-/-^ mice. A portion of these subretinal cells (about 46%) contained both macrophage/microglia marker F4/80 and RPE marker RPE65. The protein levels of TNF-α were up-regulated in the RPE/choroid and retina of old Cxcr5^-/-^ mice. Additionally, spontaneous NV-like lesions developed in the subretinal space of aged Cxcr5^-/-^ mice (S4 Fig). The numbers of eye/mice that were examined and had AMD-like pathological features were summarized in Table 1.

**Table 1:** Summary of pathology, method, and eye/mouse numbers that were examined and had age-related pathologicalchanges.

Pathology	Method	Eye/mouse #	Eye/mouse #
		(Examined)	(Pathology)
Drusen-like spots	Fundus Exam	10/5	8/4
RPE atrophy	Fundus Exam	10/5	3/3
RPE vacuoles	H&E stain	5/5	4/4
drusen-like deposits	H&E stain	5/5	3/3
NV-like lesion	H&E stain	5/5	2/2
RPE basal deposits	TB stain	5/5	2/2
Lipid droplets	ORO stain	5/5	3/3
Lipofuscin	FM	5/5	3/3
ZO-1 degradation	WB & IF	5/5	3/3
Phr apoptosis	TUNEL	5/5	3/3
Subretinal immune cells	IF & FM	5/5	3/3

17-month-old Cxcr5^-/-^ mice were used for calculation. GA: geographic atrophy. ORO: oil red O. NV-neovascularization. FM: fluorescence microscope. IF: immunofluorescence. Phr: photoreceptor. RPE: retinal pigment epithelium. TB: toluidine blue. WB: Western blots.

Despite these findings some questions remain to be addressed. First, are the white spots in aged Cxcr5^-/-^ mice similar to the drusen in AMD patients? Lipids and lipofuscin deposits in the subretinal space, RPE and choroid may contribute to these deposits, which were observed with the retinal fundus imaging. Another contributor might be the increased microglia/macrophage that accumulated to the sub-retinal space, as was shown in aged Cx3cr1^-/-^ mice [22]. Additionally, the sub-RPE deposits were observed with light microscope and TEM (Figs 3B and 4C). More importantly, the dome-shaped hard drusen-like deposits were identified on cross sections (Fig 3A). Also, whether they are comparable drusen in human AMD needs to be determined. The molecular compositions of drusen in AMD subjects, such as CFH, apoE and crystalline [50] can be used for further characterization (e.g., by immunohistochemistry and immunofluorescence). Second, what are the mechanisms regulating retinal cell death (e.g., photoreceptor and RPE) in aged Cxcr5^-/-^ mice? Apoptosis is known to regulate photoreceptors and RPE cell death in dry AMD. Recent studies have shown that programmed necrosis or necroptosis mediated by TNF-α and RIPK3 is the underlying mechanism for RPE death induced by double strand RNA *in vivo* [49] and oxidative stress *in vitro* [51]. It remains unclear whether apoptosis, necropotosis, or both control RPE death in the aged Cxcr5^-/-^ mice. Third, are complement and inflammasome activated? And if so, how much do they contribute to the AMD pathogenesis in aged Cxcr5^-/-^ mice? Genetic analysis (e.g., CFH gene) studies [7–9] have revealed that the activation of complement alternative pathway significantly contributes to the pathogenesis of AMD. Inflammasome mediated by Caspase1-NRLP3 signaling pathway is activated in the RPE death elicited by Alu RNA accumulation caused by DICER1 gene mutation [52]. Elucidating whether these two inflammatory cascades play roles in the development of AMD-like features in aged Cxcr5^-/-^ mice will give insights into the roles of chemokine receptors in RPE death and AMD pathogenesis. Last, why were a large number of melanosomes and phagosomes present inside RPE in aged Cxcr5^-/-^ mice? Is this phenomenon only a consequence of or secondary effect to RPE death? Or is Cxcr5 necessary for phagocytotic clearance and recycling of the photoreceptor outer segment and autophagy by RPE cells? Answers to these questions are important for understanding the roles of Cxcl13-Cxcr5 signaling pathway in the pathogenesis of AMD.

Similar to Ccl2-Ccr2 and Cx3cl1-Cx3cr1 signaling pathways [25, 53], Cxcl13-Cxcr5 pathway regulates the migration of inflammatory cells, such as macrophage and microglial cells. Cxcr5 gene deficiency in senescent mice led to increased sub-retinal retention of these cells (Fig 8). It is interesting that some of these immune cells were also positive for RPE marker RPE65. Although why these sub-retinal immune cells had RPE marker is to further be elucidated, there are two postulated reasons: 1) the subretinal immune cells were transformed from RPE, which migrated to the subretinal space; 2) they were infiltrated immune cells (e.g., macrophage) and phagocytized the damaged or dead RPE cells, leading to some subretinal immune cells with the enclosed RPE markers. It is important to note that the functional role of Cxcr5 may be distinct between aged eye and laser-induced CNV: protective in the former, but pro-angiogenic in the latter. In the laser-induced CNV, the Cxcr5-expressing immune and inflammatory cells infiltrated to the sub-retina and CNV (unpublished results). Some Cxcr5^+^ cells were positive for Iba1, CD11b, or CD45, indicating they were macrophage and microglia cells. However, others were negative for Iba1 or CD11b, suggesting that Cxcl13-Cxcr5 signaling axis may regulate the migration of other immune cells. It is possible that the Cxcr5^+^CD45^+^Iba1^-^CD11b^-^ cells were lymphocytes because Cxcr5 is known to regulate the trafficking of T-cells and B-cells. The functions of the infiltrated immune and inflammatory cells in the laser-induced CNV and aged eye may be disparate. In the laser-induced CNV model, these cells are likely pro-inflammatory and/or angiogenic, producing cytokines, chemokines, angiogenic and/or growth factors that are conducive to CNV. However, in aged eyes, they likely act as phagocytic cells and participate in the clearance of lipid, metabolic wastes, and other by-products from the RPE and photoreceptors. Cxcl13-Cxcr5 signaling axis may play a role in regulating the trafficking of these cells from/to the eye. Cxcr5 deficiency can impair their migrations, resulting in their sub-retinal accumulations. These accumulated inflammatory cells can initiate pathological cascades of AMD in several alternative ways: 1) elicit innate immune responses, such as complement and inflammasome activation; 2) produce pro-inflammatory cytokines, such as TNF-α and interleukin-1β; and 3) increase inflammatory cell debris, lipid deposition and oxidative stress.

In conclusion, our findings suggest that Cxcr5 itself may protect RPE and retinal cells from degeneration during aging and, therefore, its loss may be implicated in age-related pathologies, such as AMD. The mechanisms include increased inflammation, such as the accumulation of immune and inflammatory cells to the sub-retinal space and increased TNF-α expression. Further elucidation of the mechanisms can not only provide insights into AMD etiology, but help to design new therapeutic treatments for the disease.

## Supporting information

S1 Fig The specificity of Anti-Cxc5 antibody. **(A)** Western blots (WB) detected a protein band of approximately 46 kDa (the predicated molecular weight for Cxcr5 is 42 kDa). The total proteins were prepared from the retinas of C57BL/6 wild type mice at ages of 2 mo (lanes 1–3), 15 mo (lanes 4–6), and 22 mo (lanes 7 and 8). (**B and C**) Immunofluorescence staining images with the retinal sections of 15-month-old C57BL/6 wild type (**B**) and Cxcr5^-/-^ (**C**) mice. GCL: ganglion cell layer; IPL: Inner plexiform layer; INL: inner nuclear layer; OPL: outer plexiform layer; ONL: outer nuclear layer. Scale bar: 50 μm.
(TIF)

S2 Fig Glial cells express Cxcr5 in aged mouse retina. The 22-month-old C57BL/6 wild type mice were used for all the immunofluorescence staining. **(A-C)** Double labeling of Cxcr5 (green) and Lectin (red). **(D-F)** Double labeling of Cxcr5 (green) and CD11b (red). **(G-I)** Double labeling of Cxcr5 (green) and GFAP (red). GCL: ganglion cell layer; IPL: Inner plexiform layer; INL: inner nuclear layer; OPL: outer plexiform layer; ONL: outer nuclear layer. Scale bar: 50 μm.
(TIF)

S3 Fig Retinal Müller cells express Cxcl13. **(A)** Immunofluorescence staining of anti-Cxcl13 with adult (2 months) C57BL/6 wild type mouse retina. **(B)** Immunofluorescence staining of anti-Cxcl13 with aged (15 months) Cxcr5^-/-^ mouse retina. **(C-E)** Double immunofluorescence staining of anti-Cxcl13 (**C**) and anti-glutamine synthetase (GS) (**D**) with aged (15 months) C57BL/6 wild type mouse retina. The merged image (**E**) shows the co-localization of Cxcl13 and GS at GCL and IPL. GCL: ganglion cell layer; IPL: Inner plexiform layer; INL: inner nuclear layer; OPL: outer plexiform layer; ONL: outer nuclear layer. Scale bar: 50 μm.
(TIF)

S4 Fig Spontaneous neovascularization (NV)-like lesion in aged (17 months old) Cxcr5^-/-^ mice. **(A)** H&E stained sections. Arrow indicated the NV-like lesion in the subretinal space. **(B)** Immunofluorescence staining image of anti-Collagen IV (Col IV). The NV-like lesion in subretinal space was immunopositive for Col IV. **(C)** The merged picture of lectin staining image and the differential interference contrast (DIC) one. The NV-like lesion had a subretinal localization. **(D-F)** Double immunofluoresence staining sample image of anti-CD31 and anti-CD45. DAPI (blue) acted as couterstain. GCL: ganglion cell layer; IPL: inner plexiform layer; INL: inner nuclear layer; OPL: outer plexiform layer; ONL: outer nuclear layer; PS: photoreceptor segment; RPE: retinal pigment epithelium; Cho: choroid. Scale bar: 50μm.
(TIF)
