# Supplementary figures and images for: Age-related macular degeneration phenotypes are associated with increased tumor necrosis-alpha and subretinal immune cells in aged Cxcr5 knockout mice
Source: PLoS One. 2017 Mar 10;12(3):e0173716. doi: 10.1371/journal.pone.0173716 (PMC5345864; doi:10.1371/journal.pone.0173716)

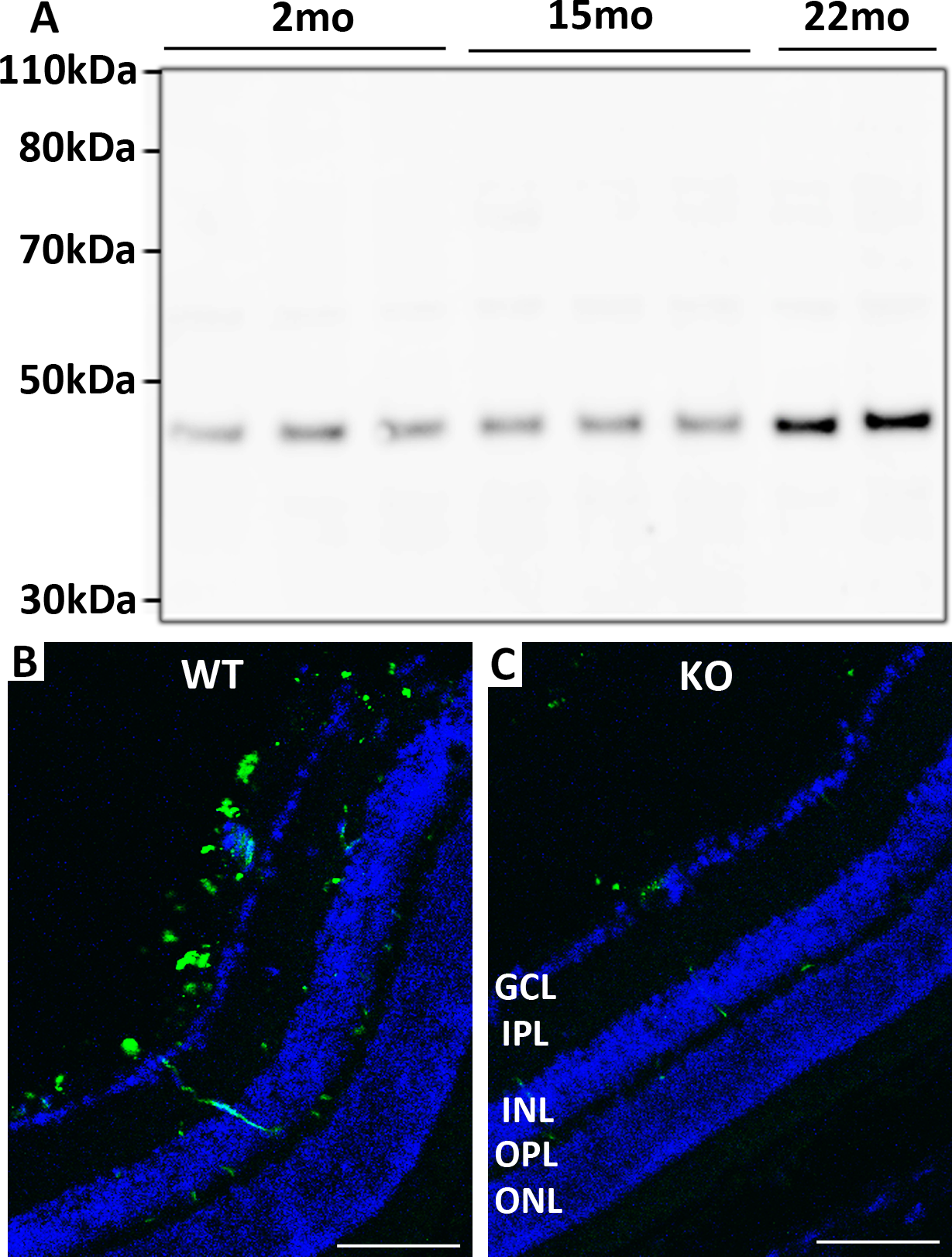

Supplement: S1 Fig — (A) Western blots (WB) detected a protein band of approximately 46 kDa (the predicated molecular weight for Cxcr5 is 42 kDa). The total proteins were prepared from the retinas of C57BL/6 wild type mice at ages of 2 mo (lanes 1–3), 15 mo (lanes 4–6), and 22 mo (lanes 7 and 8). (B and C) Immunofluorescence staining images with the retinal sections of 15-month-old C57BL/6 wild type (B) and Cxcr5-/- (C) mice. GCL: ganglion cell layer; IPL: Inner plexiform layer; INL: inner nuclear layer; OPL: outer plexiform layer; ONL: outer nuclear layer. Scale bar: 50 μm. (TIF) [file pone.0173716.s001.tif]

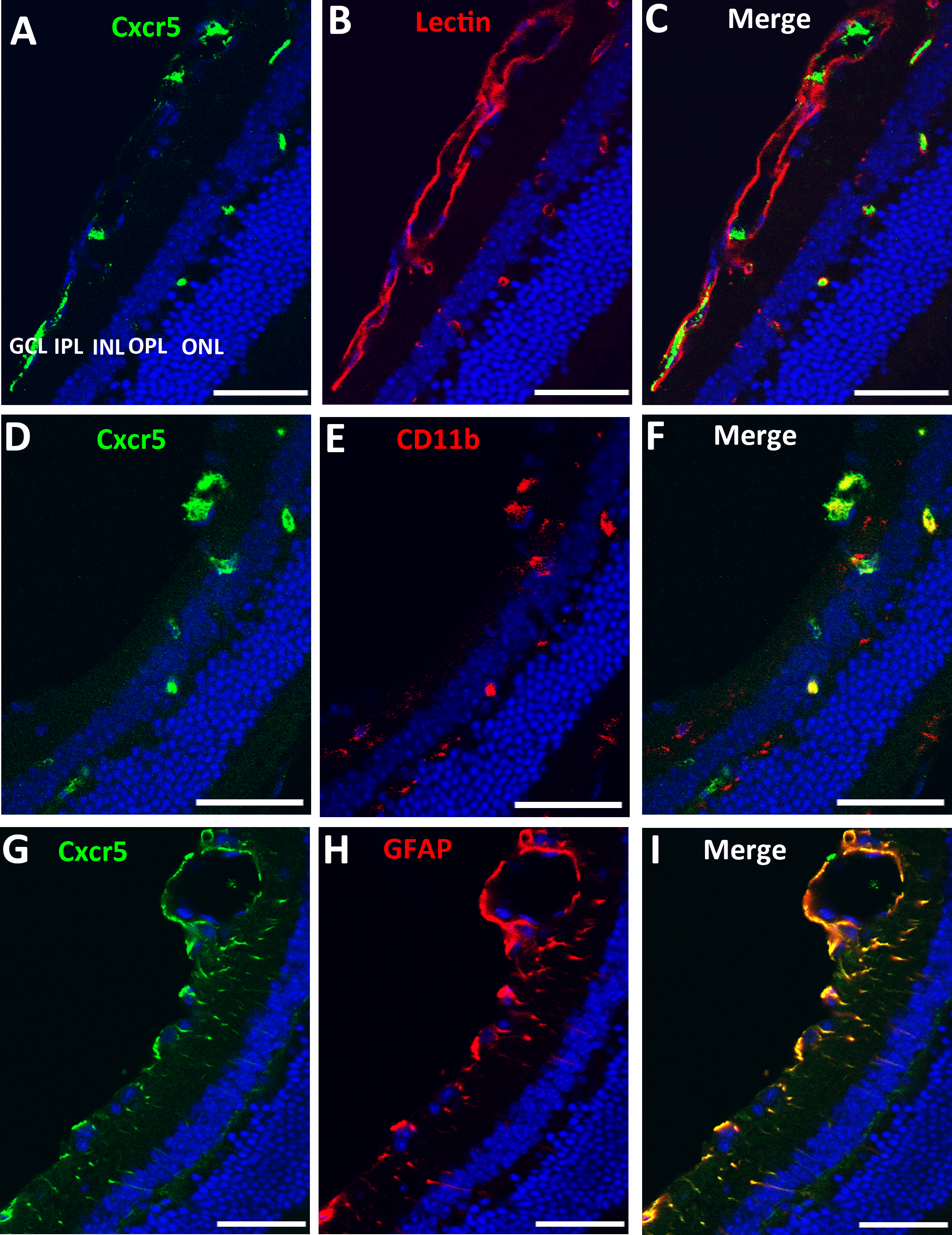

Supplement: S2 Fig — The 22-month-old C57BL/6 wild type mice were used for all the immunofluorescence staining. (A-C) Double labeling of Cxcr5 (green) and Lectin (red). (D-F) Double labeling of Cxcr5 (green) and CD11b (red). (G-I) Double labeling of Cxcr5 (green) and GFAP (red). GCL: ganglion cell layer; IPL: Inner plexiform layer; INL: inner nuclear layer; OPL: outer plexiform layer; ONL: outer nuclear layer. Scale bar: 50 μm. (TIF) [file pone.0173716.s002.tif]

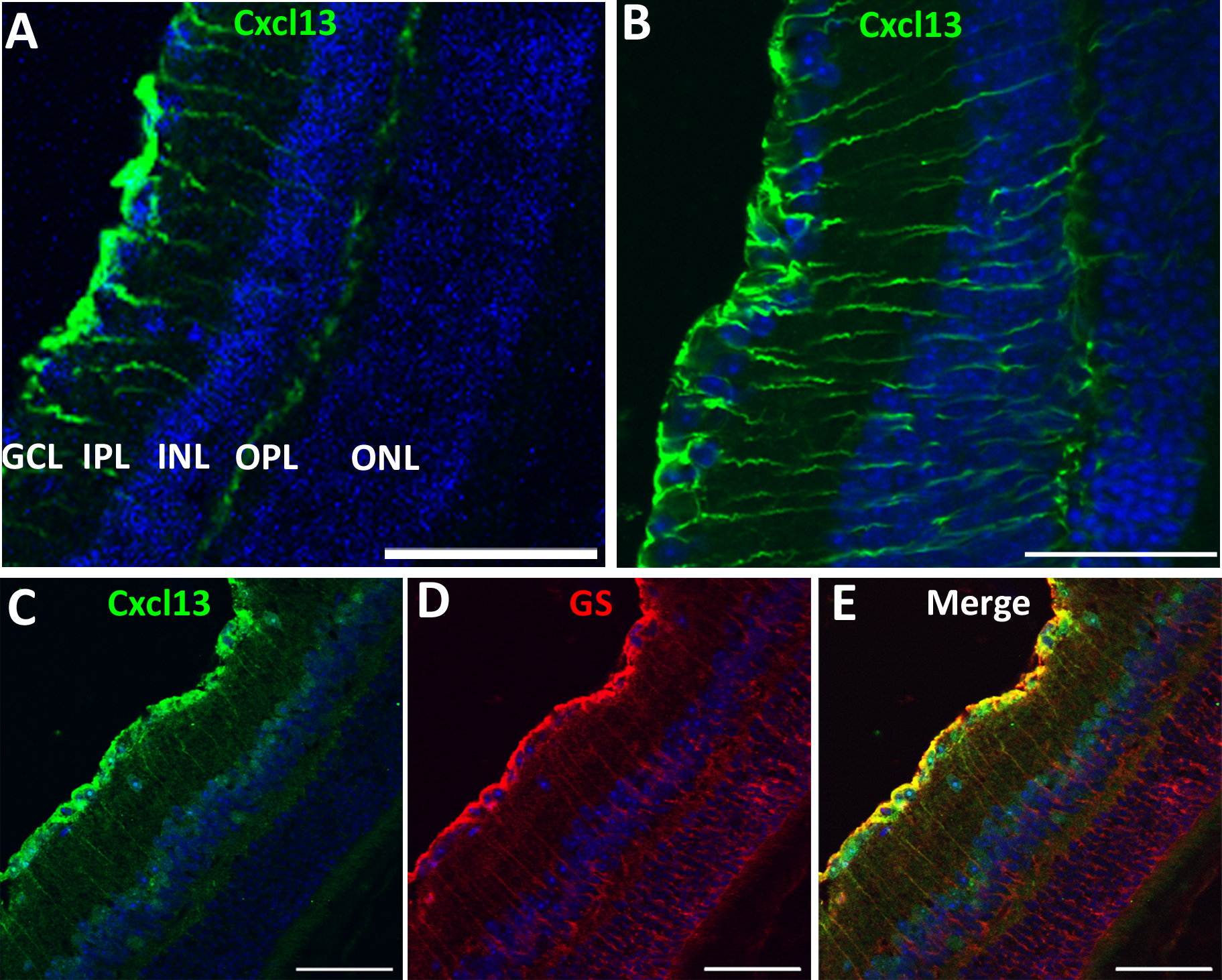

Supplement: S3 Fig — (A) Immunofluorescence staining of anti-Cxcl13 with adult (2 months) C57BL/6 wild type mouse retina. (B) Immunofluorescence staining of anti-Cxcl13 with aged (15 months) Cxcr5-/- mouse retina. (C-E) Double immunofluorescence staining of anti-Cxcl13 (C) and anti-glutamine synthetase (GS) (D) with aged (15 months) C57BL/6 wild type mouse retina. The merged image (E) shows the co-localization of Cxcl13 and GS at GCL and IPL. GCL: ganglion cell layer; IPL: Inner plexiform layer; INL: inner nuclear layer; OPL: outer plexiform layer; ONL: outer nuclear layer. Scale bar: 50 μm. (TIF) [file pone.0173716.s003.tif]

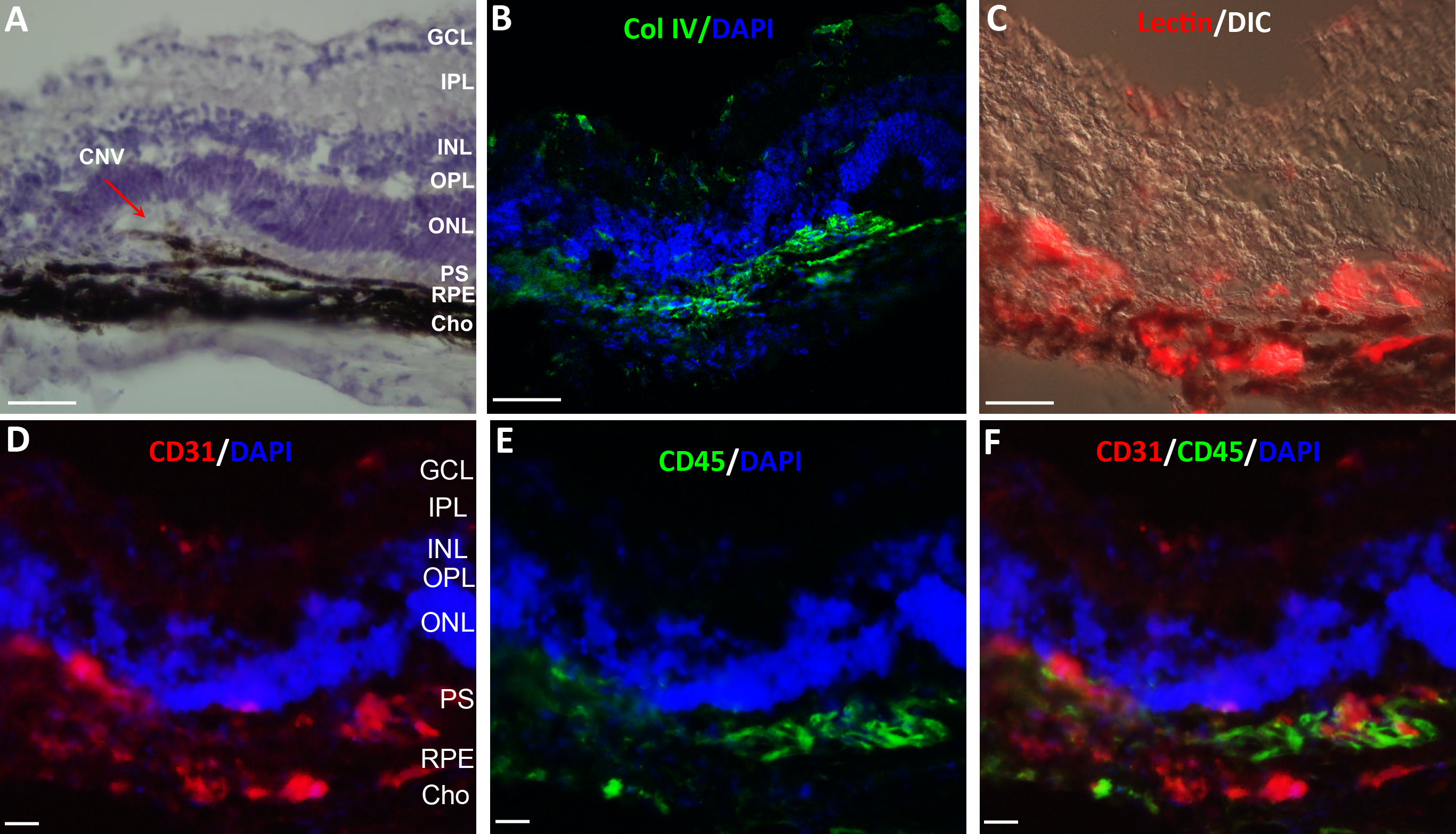

Supplement: S4 Fig — (A) H&E stained sections. Arrow indicated the NV-like lesion in the subretinal space. (B) Immunofluorescence staining image of anti-Collagen IV (Col IV). The NV-like lesion in subretinal space was immunopositive for Col IV. (C) The merged picture of lectin staining image and the differential interference contrast (DIC) one. The NV-like lesion had a subretinal localization. (D-F) Double immunofluoresence staining sample image of anti-CD31 and anti-CD45. DAPI (blue) acted as couterstain. GCL: ganglion cell layer; IPL: inner plexiform layer; INL: inner nuclear layer; OPL: outer plexiform layer; ONL: outer nuclear layer; PS: photoreceptor segment; RPE: retinal pigment epithelium; Cho: choroid. Scale bar: 50μm. (TIF) [file pone.0173716.s004.tif]
